# First record of the genus *Empidideicus* Becker, 1907 (Diptera, Mythicomyiidae) in China and the Oriental Region, with description of a new species

**DOI:** 10.3897/zookeys.846.30391

**Published:** 2019-05-16

**Authors:** Gang Yao, Gao Chen

**Affiliations:** 1 Jinhua Polytechnic, Jinhua, Zhejiang 321007, China Jinhua Polytechnic Jinhua China; 2 Yunnan Key Laboratory for Integrative Conservation of Plant Species with Extremely Small Populations, Kunming Institute of Botany, Chinese Academy of Sciences, Kunming, 650204, Yunnan, China Kunming Institute of Botany, Chinese Academy of Sciences Kunming China

**Keywords:** *
Empidideicus
*, flower visiting, new record, new species, pollinator

## Abstract

*Empidideicus* Becker, 1907 is newly recorded from China and the Oriental Region, with one new species, *E.pentagonius***sp. n.**, described and illustrated. Observations are provided on the biology of *E.pentagonius***sp. n.** visiting flowers of *Stemonamairei* (Levl.) Krause (Liliflorae, Stemonaceae). A key to the genera of Mythicomyiidae known to occur in China is provided.

## Introduction

Mythicomyiidae is a cosmopolitan family in the Bombylioidea with more than 330 described species in 25 extant genera from six subfamilies (Evenhuis 2002). This family has the greatest diversity in semi-arid and arid regions and is strongly associated with flowers. Hitherto, only three genera of Mythicomyiidae have been reported from China: *Mythenteles* Hall & Evenhuis, 1986, *Cephalodromia* Becker, 1914, and *Platypygus* Loew, 1844.

*Empidideicus* Becker, 1907 belongs to the monogeneric subfamily Empidideicinae. So far, 42 species have been described, with 20 species distributed exclusively in the Afrotropical Region and 20 species distributed exclusively in the Palaearctic Region, and two species are distributed in both Afrotropical and Palaearctic regions (Evenhuis 2002, 2007, 2009; Gharali et. al. 2010, 2011, 2015; Hakimian et. al. 2014). The tribe Empidideicini was established by Hull (1973) within the Mythicomyiinae, when he first introduced tribal-level classification into Bombyliidae. Initially four genera, *Empidideicus* Becker, 1907, *Anomaloptilus* Hesse, 1938, *Euanthobates* Hesse, 1965, and *Leylaiya* Efflatoun,1945, were included in Empidideicini. Recent studies have focussed on the Afrotropical fauna (Evenhuis 2009, 2007), and described 10 new species of *Empidideicus* in Iran (Gharali et. al. 2010, 2011, 2015; Hakimian et. al. 2014).

The genus *Empidideicus* is reported from China and Oriental Region for the first time, and a new species, *E.pentagonius* sp. n., is described. Observations are provided on the flower visiting behaviour of *E.pentagonius* in northwestern Yunnan, China. A key to the genera of Mythicomyiidae from China is presented. The distribution of the new species updates the easternmost distribution of the genus and more species might be distributed in the dry-hot valleys of the Oriental and eastern Palaearctic regions.

## Material and methods

Specimens were collected by sweeping flowers of *Stemonamairei* (Levl.) Krause in June beside the Jinsha River in southwest China (28°21'18.91"N, 99°12'52.20"E). The photos of adults visiting flowers were taken with a Canon 5D digital Camera and combined into figures using Adobe Photoshop CS3 software. Photos of male genitalia were taken by KEYENCE VHX-2000. The specimens were studied and illustrated with an Olympus SZ61 stereo microscope. Preparations of genitalia were made by macerating the apical portion the abdomen in cold 10% NaOH for 12–15 h. After examination, dissected material was transferred to fresh glycerine and stored in a microvial together with the specimen. The holotype and other specimens examined are deposited in the Entomological Museum of the China Agricultural University, Beijing (CAU).

## Taxonomy

### Key to genera of Mythicomyiidae from China

**Table:** 

1	Wing vein R_2+3_ absent	**2**
–	Wing vein R_2+3_ present	**3**
2	Wing m-cu crossvein present; female spermathecae spherical with apical invagination	*** Empidideicus ***
–	Wing m-cu crossvein absent; female spermathecae reservoir conical	*** Mythenteles ***
3	Wing cell dm open distally, not closed by crossvein	*** Cephalodromia ***
–	Wing cell dm closed distally by crossvein	*** Platypygus ***

### Genus *Empidideicus* Becker

*Empidideicus*Becker 1907: 97. Type species: *Empidideicuscarthaginiensis* Becker, 1907, by monotypy.

*Cyrtoides*Engel 1933: 102 (as subgenus of *Empidideicus* Becker). Type species: *Empidideicusefflatouni* Engel, 1933, by monotypy.

*Ecliptica*Engel 1933: 103. Unavailable name; name proposed in synonymy with *Cyrtoides* and not made available before 1961.

*Anomaloptilus*Hesse 1938: 983 (as subgenus of *Empidideicus* Becker). Type species: *Empidideicuscelluliferus* Hesse, 1938, by monotypy.

*Aetheoptilus*Hesse 1967: 112 (as subgenus of *Empidideicus* Becker). Type species: *Empidideicuszuluensis* Hesse, 1967, by original designation.

#### Empidideicus pentagonius sp. n.

Taxon classificationAnimaliaDipteraBombyliidae

http://zoobank.org/5BA3F89D-BC77-4EA0-A116-CAE9501157D5

Figures 5 , 6–9 , 10–11

##### Diagnosis.

Head with ocellar tubercle yellowish, frons and face yellowish with a cup-shaped brown area between frons and face; thorax with two yellowish subtriangular marks anterolaterally, with a subtrapezoidal yellowish brown area posteriorly; katepisternum with upper 1/3 yellow; aedeagal apodeme base semicirclular, with acute tip in dorsal view, aedeagal apodeme arched in lateral view; epiphallus pentagonal, with narrow tip in dorsal view.

**Figure 1.**
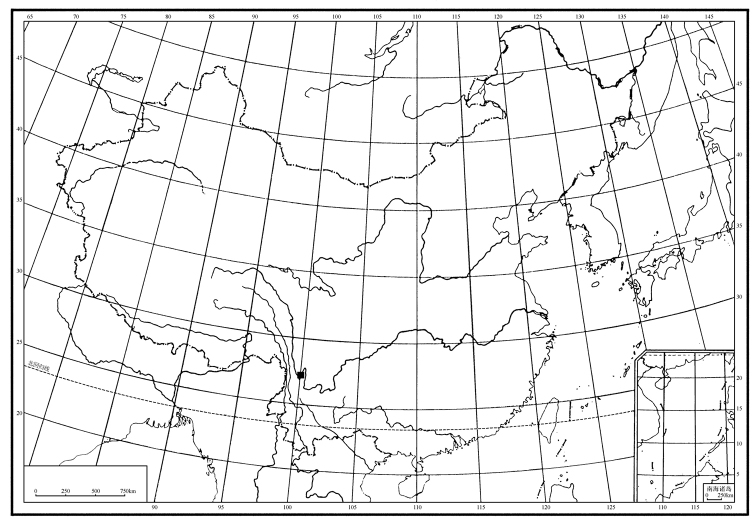
The location of the specimens of *Empidideicuspentagonius* sp. n. collected.

**Figures 2–5.**
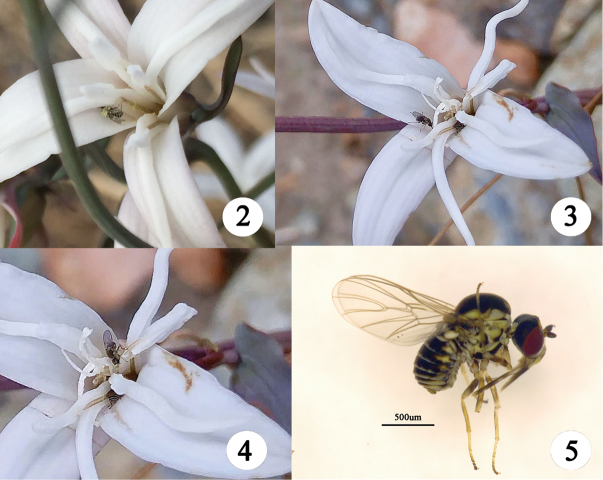
**2–4** Photographs of *Empidideicuspentagonius* sp. n. visiting *Stemonamairei***5** adult of *Empidideicuspentagonius* sp. n. Photographs in nature by Yan Qin.

##### Description.

Male. Body length 0.8–1.4 mm, wing length 1.1–1.4 mm.

Head black and yellowish, eyes red, bare; ocellar tubercle black, ocelli yellowish; eyes dichoptic, 2 × width of ocellar tubercle, frons and face bare, yellowish, except a cup-shaped brown area between frons and face; occiput black. Antenna (Fig. 11) yellowish brown, scape semicircular nearly twice wider than long; pedicel trapezoidal, slight wider than long; first flagellomere ovoid, nearly 1.7 × longer than wide; second flagellomere about 1/3 length of first flagellomere, cylindrical, about 3 × longer than wide, with minute apical style. Antennal ratio: 1:2:8. Proboscis brown except base with a yellowish quadrilateral area laterally, nearly 2 × length of head.

Thorax (Fig. 10) black and yellowish, mesonotum mostly black except edge yellowish, postpronotal lobe yellowish, anterior with two yellowish subtriangular marks laterally, and a subtrapezoidal yellowish brown area posteriorly, mesonotum with three brown prealar bristles, anepisternum and anepimeron mostly yellow except edge of front and bottom black, katepisternum mostly black except upper 1/3 yellow.

Scutellum yellowish brown. Legs yellow except femora and tarsi brown. Legs with short brown hairs; tibiae with short black hairs and bristles, tarsi with short black hairs.

Wing (Fig. 9) hyaline, except veins brown. Wing length 2.3 × width, wing with veins R_1_, R_4+5_, M_1_, M_2_, M_1+2_, CuA and Cup present, Sc incomplete; Costa, Sc, R_4+5_ and CuA_2_ strongly sclerotized, vein M_1_, M_2_, M_1+2_ and CuA_1_ less sclerotized; vein R_1_ ending nearly in middle of costa, R_4+5_ slightly curved anteriorly, M_1_ and M_2_ form an acute angle, crossvein r-m at bottom of cell dm. Wing with tiny hairs at margin. Halteres yellowish, except edge of tip brown.

Abdomen with all tergites dark brown, except posterior margin with narrow pale brown band, and with yellow posterolaterally. Sternites yellowish mostly, except yellowish brown centrally, and pale laterally.

**Figures 6–9.**
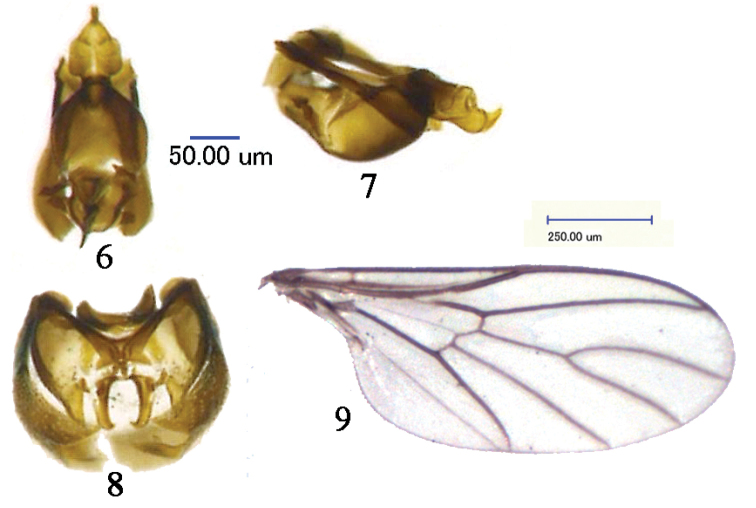
Photographs of male genitalia and wing of *Empidideicuspentagonius* sp. n. **6** phallus and gonocoxa, dorsal view **7** phallus and gonocoxa, lateral view **8** epandrium, ventral view **9** wing.

Male genitalia brown and black (Figs 6–8). Epandrium brown except edge black, nearly as long as wide, cercus well exposed, narrow and long, tip acutely in ventral view; gonocoxa L-shaped, nearly 2 × longer than wide, with acute tip, gonostylus triangular with acute tip in lateral view; aedeagal apodeme base semicircle, extremely long, and narrowly apically, with acute tip in dorsal view, aedeagal apodeme arch in lateral view; epiphallus pentagonal, with narrow tip in dorsal view, epiphallus tip sickle-shaped in laterally view.

**Figures 10, 11.**
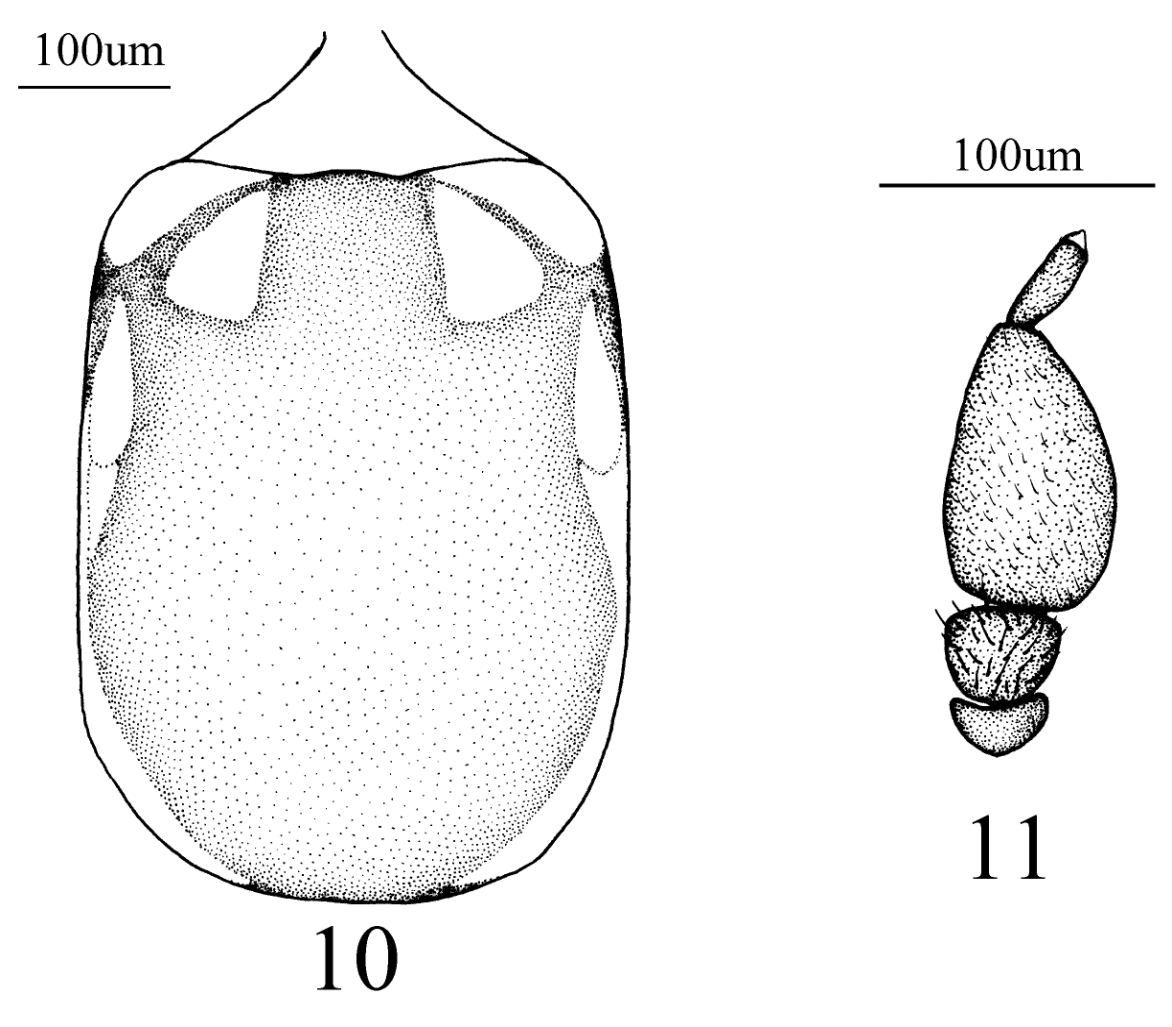
*Empidideicuspentagonius* sp. n. **10** Thorax, dorsal view **11** Antenna, dorsal view.

**Figures 12, 13.**
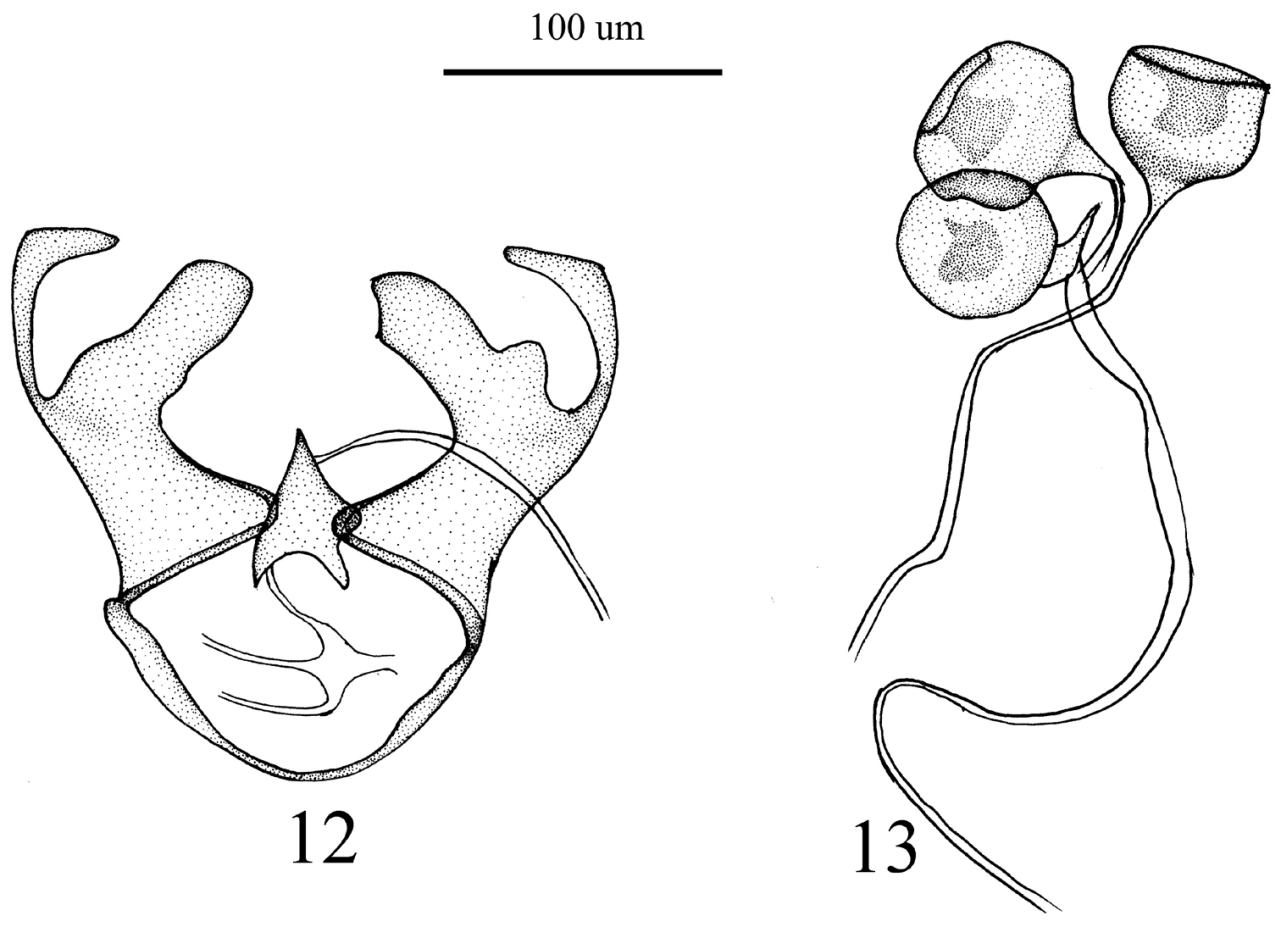
Female genitalia of *Empidideicuspentagonius* sp. n. **12** Female genitalia **13** spermathecal bulb.

Female. Body length 1.2–1.7 mm, wing length 1–1.2 mm. Female genitalia (Figs 12, 13) furca subtriangular, 1.7 higher than wide, with concavity at middle of bottom; spermathecal bulb subglobular when viewed on edge, nearly rectangular in lateral view, invaginated apically, subquadrate in form, slightly wider than deep in lateral view.

##### Type material.

Holotype male, CHINA: Yunnan Deqin Benzilan (28°21'18.91"N, 99°12'52.20"E), 08–18. XI. 2017, Yan Qin; Paratype female, CHINA: Yunnan Deqin Benzilan (28°21'18.91"N, 99°12'52.20"E), 08–18. XI. 2017, Yan Qin; 29 males 12 females, CHINA: Yunnan Deqin Benzilan (28°21'18.91"N, 99°12'52.20"E), 08–18. XI. 2017, Yan Qin.

##### Distribution.

China (Yunnan).

##### Etymology.

The specific name refers to the epiphallus pentagonal in dorsal view.

##### Remarks.

The new species is similar to *E.legulicoxa* Gharali & Evenhuis, 2010 (Iran), but it can be separated from the latter by the following features: the frons and face are yellowish, except a cup-shaped brown area between frons and face; the katepisternum mostly black except for the upper 1/3, which is yellow; the abdomen with all tergites is dark brown, except the posterior margin, which has a pale brown narrow band, and laterally, which is yellow posteriorly. In *E.legulicoxa*, the frons is yellowish white, and slightly depressed medially with a large squarish brown spot medially, and the lower 3/4 of the katepisternum is yellowish white; the abdomen is predominantly yellow and with medial brown color dorsally, tergites I–III are brown with undulating posterior margins (Babak et al. 2010).

## Observations

*Empidideicuspentagonius* sp. n. is one of the most important pollinators for *Stemonamairei* (Levl.) Krause in southwestern China (Fig. 1) (Yan Qin personal observation). During the collection of the specimens, the following observations were made by Yan Qin (Yan Qin 2018): (1) *E.pentagonius* sp. n. rests on the flowers of *Stemonamairei* for a few minutes to half an hour, apparently feeding pollen or nectar. (2) After visiting a flower, much pollen was observed on the body of flies, which visited one flower after another. (3) *E.pentagonius* sp. n. is considered an important pollinator of *S.mairei* in June, but this species is rare in July, and beetles became the dominant visitors of *S.mairei* instead. (4). Different from other species of *Stemona*, *S.mairei* has a faint fragrance instead of a rotting smell (Chen et. al. 2017), which might attract *E.pentagonius*. (5) The eggs and larvae of the flies were not found in the flower, and the life history of *E.pentagonius* is unknown (Figs 2–5).

## Acknowledgements

We are very grateful to Yan Qin (Kunming) for collecting the specimens. Our thanks also go to Dr Babak Gharali (Tehran) and Dr Neal Evenhuis (Honolulu) for providing important references and for their great help in the study. Thanks to Xuankun Li (Canberra) for comments on a draft of the paper, as well as Allan Cabrero (Berkeley) and Xuankun Li (Canberra) for suggestions and comments on a draft of the paper. Support for this study was provided by grants from the NSFC-Yunnan joint fund to support key projects to G. Chen (grant no. U1602264) and National Natural Science Foundation of China (no. 31301670) as well as China Postdoctoral Science Foundation to Gang Yao (no. 2015M581205).

## Supplementary Material

XML Treatment for Empidideicus pentagonius
